# Roles of the Picornaviral 3C Proteinase in the Viral Life Cycle and Host Cells

**DOI:** 10.3390/v8030082

**Published:** 2016-03-17

**Authors:** Di Sun, Shun Chen, Anchun Cheng, Mingshu Wang

**Affiliations:** 1 Avian Disease Research Center, College of Veterinary Medicine, Sichuan Agricultural University, Wenjiang, Chengdu 611130, China; sundi0921@126.com (D.S.); sophia_cs@163.com (S.C.); 2 Institute of Preventive Veterinary Medicine, Sichuan Agricultural University, Wenjiang, Chengdu 611130, China; 3 Key Laboratory of Animal Disease and Human Health of Sichuan Province, Wenjiang, Chengdu 611130, China

**Keywords:** 3C^pro^, structure, viral replication, translation initiation, pathogenesis, protease inhibitor

## Abstract

The *Picornaviridae* family comprises a large group of non-enveloped viruses that have a major impact on human and veterinary health. The viral genome contains one open reading frame encoding a single polyprotein that can be processed by viral proteinases. The crucial 3C proteinases (3C^pro^s) of picornaviruses share similar spatial structures and it is becoming apparent that 3C^pro^ plays a significant role in the viral life cycle and virus host interaction. Importantly, the proteinase and RNA-binding activity of 3C^pro^ are involved in viral polyprotein processing and the initiation of viral RNA synthesis. In addition, 3C^pro^ can induce the cleavage of certain cellular factors required for transcription, translation and nucleocytoplasmic trafficking to modulate cell physiology for viral replication. Due to interactions between 3C^pro^ and these essential factors, 3C^pro^ is also involved in viral pathogenesis to support efficient infection. Furthermore, based on the structural conservation, the development of irreversible inhibitors and discovery of non-covalent inhibitors for 3C^pro^ are ongoing and a better understanding of the roles played by 3C^pro^ may provide insights into the development of potential antiviral treatments. In this review, the current knowledge regarding the structural features, multiple functions in the viral life cycle, pathogen host interaction, and development of antiviral compounds for 3C^pro^ is summarized.

## 1. Introduction

Members of the *Picornaviridae* family are positive-strand viruses that have a major impact on the health of humans and animals. This family is composed of 29 genera, including *Aphthovirus*, *Cardiovirus*, *Kunsagivirus*, *Enterovirus*, *Hepatovirus* and *Sicinivirus* [1,2,3]. The *Picornaviridae* family consists of at least 285 different picornaviruses that can infect various hosts, and outbreaks of several viruses have caused serious diseases and substantial economic burden [4,5,6]. This review mainly focuses on the 3C proteinases (3C^pro^s) of the *Aphthovirus*, *Enterovirus* and *Hepatovirus* genera.

**Figure 1:**
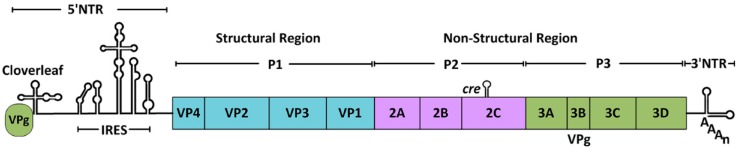
The entire genome structure of poliovirus (PV) [9]. This RNA genome contains a 5′-nontranslated region (NTR), a large open reading frame, a 3′NTR and a poly (A) tail. A small viral-encoded protein, 3B (VPg), is linked to the 5′ terminus of the RNA. The 5′NTR consists of a cloverleaf structure and a type II internal ribosome entry site (IRES). The open reading frame encodes a single polyprotein comprising the structural protein P1 region and the non-structural protein P2 and P3 regions.

Despite the diversity of picornaviruses, their genome structures and translation processes are highly conserved. Picornaviruses are small, non-enveloped viruses containing a single-stranded RNA genome with a length of 7.0–8.5 kb. The viral genome contains one open reading frame that encodes a single polyprotein comprising a structural protein P1 region and non-structural protein P2 and P3 regions. Different from cap-dependent initiation of translation, the 5′ end of picornavirus genomic RNA is linked to a small viral-encoded protein (VPg), instead of 7-methylguanosine, which is necessary for initiating viral RNA replication (Figure 1). The release of mature and functional proteins from the polyprotein is primarily mediated by viral proteinases including 3C^pro^, 2A^pro^ and leader proteinase. Most processing is performed by 3C^pro^ and the 3CD precursor.

In this review, we summarize how 3C^pro^ is involved in polyprotein processing, protein-primed RNA synthesis initiation and the switch from viral translation to replication. We discuss the multiple roles that 3C^pro^ plays in the host cells, including shutting off transcription, inhibiting protein synthesis, blocking nucleocytoplasmic transport and inducing cell death. In addition, we also compare the functions of 3C^pro^ in the pathogenesis process of different picornaviruses. Discoveries have recently been made concerning effective and broad-spectrum inhibitors of picornaviruses. As a protease inhibitor for rhinovirus (RV) 3C^pro^, rupintrivir (AG7088) has been the subject of clinical trials [7], however, AG7088 failed in a natural infection study [8]. Efforts to develop effective antiviral compounds are still ongoing. The synthesis of AG7088 analogues, the exploration of non-covalent inhibitors and research on natural medicine are the main strategies currently being used to develop 3C^pro^ inhibitors (3CPIs).

## 2. The Mechanism of Proteolysis

Although it is an unusual chymotrypsin-like cysteine protease, 3C^pro^ adopts a fold similar to that of the serine protease chymotrypsin; indeed, 3C^pro^ combines features of both serine and cysteine enzymes. Structural studies on picornaviral 3C^pro^ may identify unique features, providing useful information on protease inhibitors.

Since the 1990s, crystal structures have been determined for the 3C^pro^s of human rhinovirus (HRV), poliovirus (PV), hepatitis A virus (HAV), foot-and-mouth disease virus (FMDV) and enterovirus 71 (EV71) [10,11,12,13]. These studies have revealed two equivalent β-barrel domains in 3C^pro^ located approximately 90° from each other and composed of six antiparallel strands. Moreover, an extended shallow groove for substrate binding is located between the two domains [14]. A flexible surface loop, called the β-ribbon, has been observed in several picornaviral 3C^pro^s, including those of HRV (12 residues), PV (12 residues), and HAV (21 residues) [14,15]. The 13-residue β-ribbon of FMDV was considered in previous studies to be disordered but was subsequently demonstrated to be flexible [16]. The β-ribbon folds over the peptide-binding groove of the enzyme and accommodates a portion of the *N*-terminal peptide substrate. Thus, the β-ribbon plays a crucial role in substrate recognition. According to studies of EV71 3C^pro^, the β-ribbon is thought to alternate between an open conformation and a closed conformation with the aid of hinge residues (Gly123 and His133) [17]. Before binding a substrate, the β-ribbon adopts an open conformation to increase substrate accessibility by exposing more substrate binding clefts; once bound, the interaction between the β-ribbon and N-terminal end of the substrate stabilizes the closed conformation to form an enzyme-substrate complex.

Picornavirus 3C^pro^s possesses a conserved Cys-His-Asp/Glu catalytic triad within the active site. This structure is similar to the Ser-His-Asp catalytic triad of serine proteases, except that third residue of the triad plays a less important catalytic role than in a serine protease. The catalytic residues in HAV include Cys-172 as a nucleophile and His-44 as a general base to form a Cys-His dyad [18]. Proteolytic mechanism studies in EV71 have shown that an uncharacterized residue Arg39 is involved in the proteolysis reaction by altering the distance to Glu71 as a charge neutralizer. Arg39 residue is conserved among CVB3, simian enterovirus (SEV) and FMDV 3C^pro^ whereas a conserved substitution of R39K is present in the 3C^pro^ of porcine enterovirus (PEV). In addition, Arg39 is replaced by an uncharged threonine or serine in the 3C^pro^ of PV, HRV and HAV, thereby indicating that Arg39 cannot be a neutralizer [19]. His40, a catalytic residue of triad, adopts two conformations as follows: a productive conformation that has been observed in most picornaviral 3C^pro^s and accounts for 39%; and a nonproductive conformation in which an imidazole side chain is positioned outside the hydrogen bonding distance to Cys-147, accounting for 61%. Thus, the negatively charged carboxylate side chain of Glu71 plays a critical role in maintaining the entire architecture of the active site and in stabilizing the productive conformation of His40 [19].

The cleavage specificity of 3C^pro^ has largely been investigated via extensive sequence analysis of cleavage junctions in polyprotein and cleavage assays using protein or peptide substrates *in vitro*. Investigations have shown that the 3C^pro^ of PV preferentially cleaves polypeptides with P1-Gln/P1’-Gly junctions. With the exception of FMDV 3C^pro^, other picornaviruses have less strict cleavage, with a small residue at P1’, such as Gly, Ser, and Ala or a hydrophobic residue at P4. The 3C^pro^ of FMDV exhibits some variability at P1’, however, the probability of P1-Gln or P1-Glu is similar [20]. Specific studies on the 3C^pro^ of FMDV have shown that when P1-Gln is present, 3C^pro^ prefers large hydrophobic residues at P1 (Leu, Ile, and Thr) and P2-Lys. Nonetheless, it is possible for a small amino acid (Gly and Ser) to be strongly associated with P1-Glu. The 3C^pro^ of HAV can tolerate a large residue at P1′ and exhibits a preference for a small hydrophilic residue at P2 [21].

## 3. The Role of 3C^pro^ in the Viral Life Cycle

### 3.1. Processing of the Viral Polyprotein

**Figure 2:**
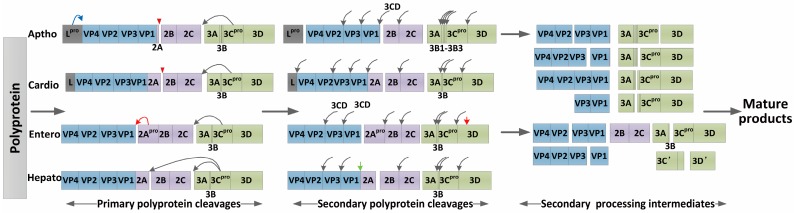
Polyprotein processing in different genera of the picornavirus family [23,28]. The release of mature and functional proteins from the polyprotein is dependent on primary and secondary cleavages and polyprotein processing differs slightly among diverse picornaviruses. In primary cleavage, L^pro^ functions in apthoviruses by freeing itself (blue arrow), whereas the cleavage of P1-P2 is mediated by ribosome skipping in apthoviruses and cardioviruses (red triangle) [29]. The proteolytic 2A^pro^ of enteroviruses is involved in primary cleavage and 3D^pol^ cleavage to produce 3D’ intermediate (red arrows). 3C^pro^ and 3CD are responsible for a proportion of primary cleavages and almost all secondary cleavages (black arrow). The hepatovirus VP1-2A cleavage can be achieved by a host cell proteinase (green arrow). See the text for details.

Multiple viral proteins are generated through primary polyprotein processing and secondary polyprotein processing, and the analysis of polyprotein cleavage has shown that 3C^pro^ is responsible for most of the cleavages among the 29 genera of the picornavirus family (Figure 2). However, several exceptions have been found at the following four cleavage sites: L-VP4, VP4-VP2, P1-2A, and 2A-2B. In aphthovirus and erbovirus, the leader protein frees itself from VP0, whereas cardiovirus, kobuvirus, teschovirus, sapelovirus and senecavirus 3C^pro^s function in the primary cleavage [22,23]. Moreover, 2A^pro^ exhibits proteinase activity or translational recoding activity during primary cleavage. The junction between P1 and 2A is cleaved by proteolytic 2A^pro^ in enteroviruses and potentially in sapelovirus type 2 (SV2) as well as PEV8. The separation of capsid proteins from replication proteins is mediated by ribosome skipping at the Asn-Pro-Gly-Pro site, inducing cleavage of 2A–2B in aphthoviruses, cardioviruses, teschoviruses, cosaviruses, sencaviruses and erboviruses [24]. Under other conditions, this cleavage is achieved by 3C^pro^ [24,25]. 3C^pro^ presents in all picornaviruses, and is responsible for almost all secondary cleavages. For Aichi virus, in which L and 2A^pro^ are not proteolytic, all cleavages are completed by only 3C^pro^ and the 3CD precursor. A series of studies on enteroviruses and apthoviruses have revealed that cleavage at VP2-VP3 and VP3-VP1 can be accomplished more efficiently by 3CD^pro^ than 3C^pro^ [26,27,28].

### 3.2. Initiation of Viral RNA Synthesis

**Figure 3:**
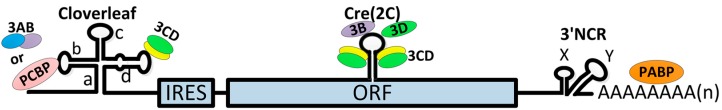
The RNA structures and proteins involved in PV RNA replication [46]. The *a, b, c* and *d* subelements form the cloverleaf structure. And the 3’NCR of RNA consists of two stem-loops (X, Y).There are three structures that can interact with 3C^pro^ and its precursor: namely the 5′ cloverleaf (5′ CL), *cis*-acting RNA elements (*cre*), and the 3′NTR poly (A). The ribonucleoprotein (RNP) complexes formed by the binding of 3CD to stem-loop *d* of 5′ CL and the binding of PCBP/3AB to stem-loop *b* are necessary for viral RNA replication. The *cre* hairpin of PV located in 2C^ATPase^ can interact with 3B, 3D^pol^ and 3CD to initiate RNA synthesis. The PABP bound to the poly (A) tail interacts with RNP for RNA replication.

Members of the picornavirus family synthesize positive- and negative-stranded RNA by relying on viral RNA-dependent RNA polymerase. The 3C^pro^ alone and its precursors, namely 3ABC, 3BC, 3BCD and P3 (3ABCD), have been demonstrated to stimulate RNA synthesis at different levels in *in vitro* systems [30,31,32,33,34]. There are three RNA structures in the genome, which are important for RNA replication and which interact with 3C^pro^ and its precursor (Figure 3) [35]. These interactions are important for RNA replication in PV. First, a cloverleaf-like structure in the 5′-NCR of the viral RNA (5′CL) is important for RNA replication [36,37] and binding between 3CD and stem-loop *d* as well as binding between poly(rc)-binding protein 2 (PCBP2) and stem-loop *b* are required for viral replication in PV [38]. Mutation (K12N/R13N) in the 3C-binding region of PV results in complete inhibition of (−)strand synthesis. Complementation assays provide evidence that P3 is the preferred precursor that interacts with 5′CL. Upon binding to RNA, P3 can recruit another P3 to release 3D and VPg for efficient replication [30]. Second, the *cis*-acting replication RNA element (*cre*) is also important for RNA replication. During initiation of RNA synthesis, *cre* interacts with two molecules of 3CD, as shown by the “slide back” model for VPgpUpU synthesis in PV, and 3CD stimulates the *cre*-templated VPg uridylylation reaction. Although less effective, the function of 3CD can also be performed by 3C^pro^, the 3BC precursor and the 3BCD precursor [31,32,33]. In FMDV, uridylylation of three VPgs can be enhanced by 3CD and in contrast to PV, the 3B_3_3C and 3B_123_3C precursors of FMDV can serve as uridylylation substrates, even without added 3C^pro^ or 3CD [39,40]. Third, the 3′NTR-poly (A) tail is important for RNA replication. Interaction among 3CD, poly (A) binding-protein (PABP) and the poly (A) tail is required for negative-strand RNA synthesis to facilitate circularization of the RNA genome [41]. There are several viral and cellular proteins that have been found to bind to the 3′NCR of picornavirus genomic RNA. For PV, the 3AB and 3CD precursors have been reported to interact with the 3′NTR of genomic RNA [42]; for HAV, the 3AB and 3ABC precursors have been demonstrated to bind to the 3′NCR of genomic RNA [34].

Taken together, 3C^pro^ and the precursors exhibit essential functions in viral replication. Does proteinase activity or RNA-binding activity of the 3C domain stimulate RNA synthesis? There are three distinct regions within 3C^pro^: the N-terminal region (K12 and R13), a central region (KFRDI86) and the *C*-terminal region (T153, G154 and K155) [43,44]. Mutation (R84S/I86A) within the 3C-binding region of PV prevents stimulation of virus synthesis [45] and mutation (K12N/R13N) within the 3C-binding region results in normal processing of viral translation but complete inhibition of (−)strand synthesis [34]. The mechanism by which 3C^pro^ is involved in RNA synthesis is closely related to its RNA-binding activity. Although both 3C^pro^ and 3CD can bind to viral RNA, there are differences. A wide range of compact conformations of 3CD induced by interaction between the 3C and 3D residues exhibit more activities than 3C^pro^ and 3D^pol^ and these interactions are conducive to the different RNA-binding activities of 3C^pro^ and 3CD [45].

### 3.3. Switch from Translation to RNA Replication

Numerous studies have focused on how picornaviruses switch from translation to replication, and the cleavage of PCBP2 by 3CD^pro^ may provide mechanism [46]. For PV, a model has been proposed in which full-length PCBP2 functions in viral translation, but the cleavage product of PCBP2 functions in RNA replication [47]. Both 3C^pro^ and 3CD^pro^ interact with phosphoinositide lipids to regulate the maturation of virus replication organelles and serve as the main protease to cleave virus and host proteins to regulate processes of both. However, the protease specificities and RNA-binding capabilities of 3C^pro^ and 3CD^pro^ are different. The proteolytic processing of the *C*-terminal end of 3C^pro^ may cause the switch of activities from 3CD^pro^ to 3C^pro^ to modulate functions during the virus cycle [48]. Above all, 3C^pro^ plays a major role in regulating translation and replication by binding to RNA sequences.

## 4. Host Cells Intervention by 3C^pro^

The cleavage of host factors by 3C**^pro^** in picornaviruses greatly alters several processes that are critical for cell viability, including transcription, initiation of protein synthesis, nucleocytoplasmic transport and growth, favoring the viral replication cycle.

### 4.1. Rapid Shut-off of Transcription in Host Cells

A rapid inhibition of RNA synthesis is observed in host cells infected by picornaviruses, which is mainly attributable to picornaviral 3C^pro^ cleavage of DNA-dependent RNA polymerases. It has been demonstrated that PV 3C^pro^ enters the nucleus via precursors and induces the cleavage of TATA-binding protein-associated factor 110 (TAF110), TATA box binding protein (TBP), cAMP response element-binding protein-1 (CREB-1), octamer binding protein-1 (Oct-1), p53 and transcription factor IIIC (TFIIIC) to shut off host cell transcription [49,50,51,52,53,54] (Table 1). In addition to these transcriptional factors associated with RNA polymerase, FMDV 3C^pro^ can also interrupt transcription by cleaving nuclear histone H3, and EV71 3C^pro^ can also cleave CstF64 of the polyadenylation machinery [55,56]. Replication of the host cell occurs in the nucleus, whereas replication of the virus occurs in the cytoplasm. Furthermore, 3C^pro^ interferes with the expression of host genes by inhibiting transcription and cap-dependent translation to provide cellular resources for viral replication.

### 4.2. Rapid Inhibition of Protein Synthesis Initiation in Host Cells

**Figure 4:**
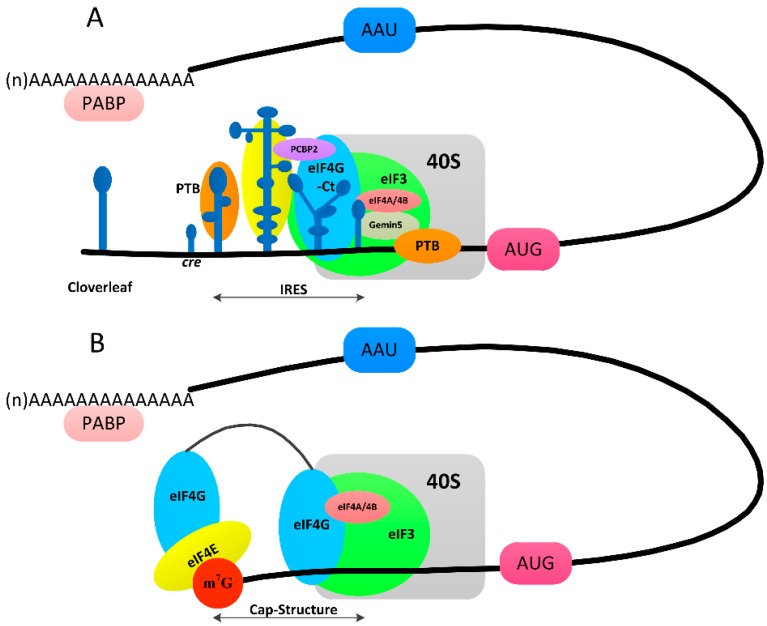
Type II IRES-dependent and cap-dependent protein synthesis initiation. (**A**) The RNA of FMDV employs a type II internal ribosome entry site (IRES) to recruit the 40S subunit to start translation. Some eIFs and ITAFs are required for IRES-driven translational initiation [60]; (**B**) The host cell employs the cap-dependent protein system. The m7G(5′)ppp(5′)N structure is recognized by a translation initiation factor (eIF4F). The binding of eIF4F to the cap and the binding of PABP to the poly (A) tail circularize the mRNA to activate it [60]. Abbreviations include: eIF4G: eukaryotic translation initiation factor 4G; eIF4E: eukaryotic translation initiation factor 4E; PTB: polypyrimidine tract-binding protein; PCBP: poly (rC)-binding protein; PABP: poly(A)-binding protein; eIF3:translation initiation factor 3; m^7^G: 7-Methylguanosine.

Protein synthesis in host cells depends on the 5′ terminal cap structure and several cap-binding proteins, but viral mRNA utilizes an IRES for translation (Figure 4). Firstly, the m7G(5′)ppp(5′)N structure is recognized by a translation initiation factor (eIF4F), which is composed of the eIF4G scaffolding protein, the eIF4E cap-binding protein and the eIF4A RNA helicase to initiate cellular mRNA translation. The 43S preinitiation complex (PIC) contains eIFs, including eIF1, eIF1A, eIF3, eIF5 and the ternary complex (eIF2-GTP-Met-tRNA), together with the 40S ribosomal subunit. The binding of eIF4F to the cap and the binding of PABP to the poly (A) tail circularizes the mRNA to activate the mRNA. The 43S ribosome then binds near the cap and scans the 5′-UTR for an AUG codon [57]. Identification of the AUG start codon constitutes the first stage of the translation of cellular mRNA (Figure 4B).

In contrast to the eukaryotic translation mechanism, picornaviruses take advantage of the IRES element to recruit ribosomal subunits for initiation [58] (Figure 4A). Reconstitution assays have indicated that assembly of 48S initiation complexes on IRES elements of types I and II (FMDV and EMCV) requires eIF3, eIF4A and the C-terminal end of eIF4G [59]. In addition to eIFs, IRES-transacting factors (ITAFs) including PTB, PCBP2, and Germin 5 contribute to the modulation of IRES activity. Furthermore, 3C^pro^ plays a significant role by cleaving some eIFs and ITAFs to guarantee the internal initiation of translation (Table 1 and Table 2).

*Picornavirus*-infected host cells experience a rapid shut-off of the cap-dependent protein and DNA repair systems. The cleavage of cellular factors as well as inhibition of their expression may explain this phenomenon (Table 2). The picornaviral proteinases, L, 2A and 3C^pro^, have been reported to primarily function in the cleavage of host translation initiation factors (eIFs). Transient expression assays have shown that eIF4A and eIF4G can be cleaved when FMDV 3C^pro^ is expressed. Furthermore, cleavage by 3C^pro^ is different from that by the FMDV leader protein and 2A^pro^ [61]. Subsequently, it has been demonstrated that L^pro^ and 3C^pro^ can function in the sequential cleavage of eIF4GI in BHK cells after infection with wild-type FMDV [62]. Two forms of eIF4A, namely, eIF4AI and eIF4AII, exist within mammalian cells. FMDV 3C^pro^ cleaves only eIF4AI, which is not related to eIF4AII even though they share 91% sequence identity [63]. In addition, during PV, CVB3 and HRV infection, eIF5B eukaryotic initiation factor is cleaved at a single site (VVEQ↓G), which separates the *N*-terminal domain from the conserved GTPase domain and *C*-terminal domain [64]. PCBP2, also known as hnRNP E2, has multiple functions in the post-transcriptional control of host and viral gene expression. The ability of PV to take advantage of PCBP2 in translation and replication has already been discussed [65], and it has also been observed that PCBP2 is cleaved by 3C^pro^ in HAV. Thus, HAV may regulate protein synthesis and RNA synthesis by 3C^pro^ [66]. In addition to PABP cleavage by 2A^pro^ and PV, HAV and EMCV 3C^pro^s have also been reported to specifically cleave PABP [67,68,69]. A study of EMCV 3C^pro^ suggests that specific PABP cleavage is required for virus replication or that full-length PABP is not absolutely required for virus replication [69]. The cleavage of Ras GTPase-activating protein-binding proteins (G3BP1) may be induced by 3C^pro^ to facilitate viral replication after CVB3 infection [70]. Recently, it has been demonstrated that the Sam68 nuclear RNA-binding protein can be cleaved by 3C^pro^ of FMDV, thus allowing 3C^pro^ to redistribute Sam68 to the cytoplasm. Remarkably, Sam68 can interact with the IRES in FMDV during infection, which then can translate viral RNA. Thus, it is speculated that Sam68 plays a supportive role in the virus life cycle [71].

### 4.3. Inhibition of Nucleocytoplasmic Trafficking by the Nuclear Pore Complex

**Figure 5:**
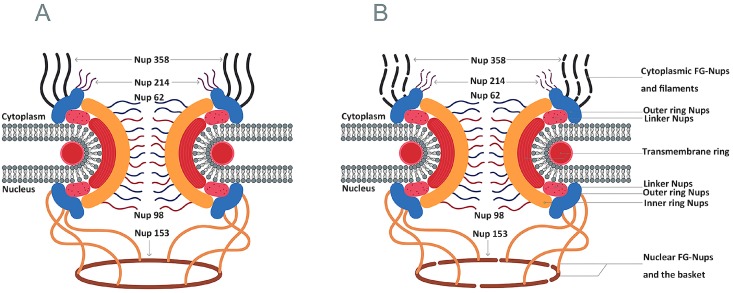
Alteration of the nuclear pore complex induced by 3C^pro^ [72,79,80]. (**A**) The nuclear pore complex is composed by the outer ring Nups, the linker ring Nups, the inner ring Nups, cytoplasmic phenylalanine-glycine (FG)-Nups, nuclear FG-Nups and the nuclear basket; (**B**) The 3C^pro^ of RV can induce cleavage of Nup 153 and Nup 214 and Nup 358.

In eukaryotic cells, DNA replication and gene transcription occur in the nucleus, whereas protein production occurs in the cytoplasm. The spatial separation of these major processes guarantees cellular transcription and translation with the help of a transport network. The nucleocytoplasmic trafficking network is responsible for transporting nuclear-resident and cytoplasmic-resident proteins between these two compartments. Translation initiation and replication in the cytoplasm depend on some nuclear-resident proteins, and picornaviruses have recently been shown to shut down the nucleocytoplasmic trafficking of cells to promote viral replication. Five nucleoporins (Nups) have been reported to be targets for picornaviral proteins to disrupt macromolecule trafficking through the nuclear pore complex (NPC) (Figure 5A). The 2A^pro^ and 3C^pro^ viral proteinases of enteroviruses are responsible for the specific proteolysis of Nup 62, Nup 98, and Nup 153, whereas the leader protein of cardioviruses induces hyper-phosphorylation of Nup 62, Nup 98, Nup 153 and Nup 214 (Table 3) [72,73,74,75]. Poliovirus 2A^pro^ causes degradation of Nup 62, Nup 98, and Nup153 in cells, and even a low concentration of 2A^pro^ can lead to Nup 98 degradation [76]. Furthermore, purified HRV 2A^pro^ cleaves Nup 62 to release FG-rich regions [77]. Expression of 3C^pro^ or 3CD in HRV 16 causes degradation of Nup 153, Nup 214 and Nup 358 in cells [78] [Figure 5B]. Moreover, the cleavage of Nup 62 is partially induced *in vitro* by a high concentration of purified HRV 3C [78]. These studies suggest that picornaviruses may normally alter nucleocytoplasmic trafficking to meet the demands of nuclear-resident proteins for viral replication but that they block the export of cellular mRNA.

### 4.4. Induction of Cell Death

Picornaviral 3C^pro^ can induce apoptosis through cleavage of transcription and translation initiation factors that control cell survival and death pathways. Expression of poliovirus 3C^pro^ results in the degradation of cellular DNA and generation of apoptotic bodies [83]. Moreover, the transcriptional activator p53 is degraded by 3C^pro^ with cellular activity [53]. p53 can also function in the cytosol and mitochondria to promote apoptosis by transcription-independent mechanisms. VAD-fmk caspase 1 inhibitor interferes with 3C^pro^-induced apoptosis [53]. For EV 71, 3C^pro^-induced apoptosis is related to the proteolytic activity of 3C^pro^, and apoptosis can be blocked by DEVD-fmk and VAD-fmk inhibitors [84]. These results suggest that caspase activation participates in the apoptotic process induced by 3C^pro.^ In addition, 2A^pro^ and 3C^pro^ induce apoptosis through multiple pathways in CVB3. Both 2A^pro^ and 3C^pro^ can induce the death receptor-mediated pathway through caspase 8-mediated activation of caspase 3 and cleavage of Bid [85]. Only 3C^pro^ can upregulate Bax and release cytochrome *c* from mitochondria in the intrinsic death pathway. Unlike these picornaviral 3C^pro^s, HAV 3C^pro^ can induce cell death independent of the caspase pathway, accompanied by cytoplasmic vacuolization [86]. Cell death is necessary for the release of virus, and 3C^pro^ influences this step through different mechanisms.

### 4.5. Other Functions

Except for their crucial role in the replication of picornaviruses and inhibition of protein synthesis in host cells, the 3C^pro^s of PV and FMDV have been previously reported to mediate cleavage of microtubule-associated protein 4(MAP-4) and cause disruption of the microtubule-organizing centre (MTOC) [87,88]. Subsequent studies have revealed that FMDV 3C^pro^ leads to fragmentation of the Golgi apparatus, which blocks the transport of proteins from the Golgi to the plasma membrane. Some scholars have studied the connection between Golgi fragmentation, microtubule depolymerization and secretion blockade. Through a series of experiments, it has been confirmed that FMDV 3C^pro^ blocks secretion in the following two ways: 3C^pro^ processes 2BC into 2B and 2C, which bind to the Golgi compartment to block intra-Golgi transport, and instead of physical binding, 3C^pro^ blocks secretion by degrading proteins necessary for intra-Golgi transports. Both of these routers depend on the proteolytic activity of 3C^pro^ [89]. Further research is needed to understand whether there is a connection between cleavage and the reduction of immune responses or between cleavage and the transmission of virus.

## 5. The Role of the Pathogenic Process

The host intrinsic immune defense against invading pathogens is closely related to the activation of parallel pattern recognition receptor (PRR) pathways that facilitate the production of type I interferon (IFN). PRRs detect conserved molecular signatures of pathogens, termed pathogen-associated molecular patterns (PAMPs), to distinguish host components from pathogens [90]. Vertebrate hosts encode several classes of PRRs, such as RIG-I-like receptors (RLRs), NOD-like receptors, C-type lectin receptors and Toll-like receptors (TLRs) [91,92]. Two well-known pattern recognition receptors are Toll-like receptors (PPRs) and RIG-I-like RNA helicases (RLHs). Notably, all TLRs are type I transmembrane proteins composed of a leucine-rich N-terminal of an ectodomain, a transmembrane domain, and a linker to a cytoplasmic *C*-terminal Toll-interleukin-I receptor (TIR) homology domain [93,94]. The RLR family, comprising RIG-I, MDA5 and LGP2, has a conserved DExD/H-box helicase domain and a C-terminal domain (CTD).

When toll-like receptor 3 (TLR3) recognizes double-stranded RNA, it recruits TIR domain-containing adapter inducing interferon-β (TRIF) [95], which together activate downstream IKK-related kinases. These kinases phosphorylate interferon regulatory factor 3 (IRF3), leading to the expression of IFN-α/β [96,97,98,99,100]. MDA5 and RIG-I, two activated DExD/H box RNA helicases, transmit signals to downstream adaptor IPS-I, leading to stimulation of NF-κB and IFN-β. The expression of IFN-α/β depends on the activation of IRF3, and this process is closely related to the interactions among IPS-I, TRF3 and TBK1/IKKi [101,102].

**Figure 6:**
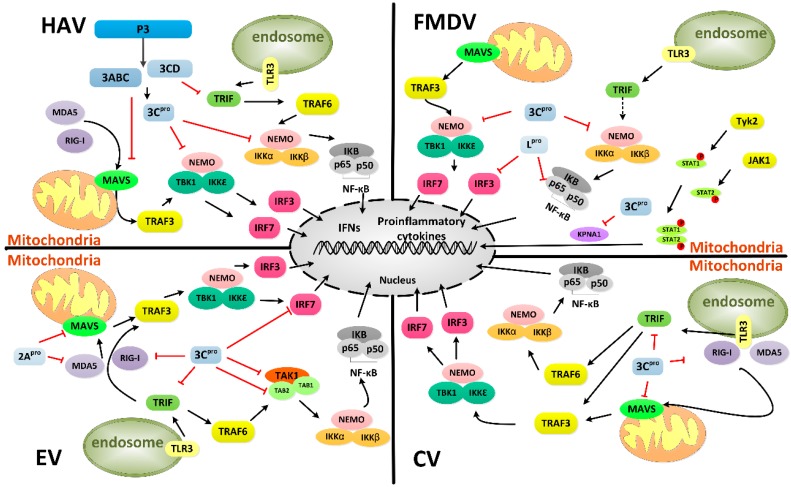
Mechanisms of 3C^pro^ from different picornaviruses suppress the immune response. It has been demonstrated that 3C^pro^ interferes with the host type I interferon (IFN) response and nuclear transcription factor kappa B (NF-κB) signaling pathway by cleaving several essential proteins. However, the mechanisms adopted by picornaviruses are slightly different. The immune response is depicted in black. Inhibition of immune response by viral proteinases is highlighted in red. Abbreviations include: MDA5: melanoma differentiation-associated gene 5; RIG-I: retinoic acid-inducible gene I; MAVS: mitochondrial antiviral signaling protein; TRIF: TIR domain-containing adapter inducing interferon-β; TLR3: toll-like receptor 3; TRAF: TNF receptor associated factor; NEMO: NF-κB essential modulator; IKK: IκB kinase; IRF: interferon regulatory factor; Tyk: tyrosine kinase; JAK1: janus kinase 1; STATs: signal transducers and activators of transcription; KPNA1: karyopherin α1.

During EV71 infection, the expression of IFN-β, tumor necrosis factor (TNF-N), and IFN-stimulated gene 54(ISG54) is inhibited. Among EV71 proteins, 3C^pro^ blocks the virus-induced activation of IFN-β by targeting the cytosolic helicase I RIG-I. However, 3C^pro^ exerts no inhibitory activity against IFN-β promoter activation, which is mediated by MDA5 in EV71 infection [92]. Enteroviral 2A^pro^ proteolytically targets MDA5 and MAVS to block the expression of IFN-I [103]. Notably, members of picornavirus family modulate MDA5 and RIG-I differently. RV and echovirus target RIG-I, whereas PV and EMCV mediate RIG-I and MDA5 [104,105,106]. Subsequent studies have revealed that EV71 3C^pro^ induces TRIF cleavage to inhibit NF-κB and IFN-β promoter activation in a caspase-independent manner (Figure 6) [107]. The use of 3C^pro^ mutants has shown that the Q312–S313 junction within TRIF might be a preferred cleavage site [104]. With regard to the constitutive activation domain of IRF7, EV71 3C^pro^ mediates cleavage of IRF7, rather than IRF3, at the Q189–S190 junction when expressed in mammalian cells (Table 4) [108]. EV71 3C^pro^ also inhibits NF-κB via cleavage of the transforming growth factor-β-activated kinase 1 (TAK1) and TAB2 complex [109]. The type I IFN antiviral response is closely related to the JAK-STAT signaling pathway, consisting of Janus kinase (JAK) family members and the signal transducers and activators of transcription (STAT) family. EV71 inhibits the type I IFN response through another mechanism of JAK1 downregulation. However, roles for the viral proteinases 2A^pro^ and 3C^pro^ as antagonists have been ruled out [110]. Recently, it has been shown that the PARP9-DTX3L complex which is formed by poly (ADP-ribose) polymerase PARP9 and the DTX3L E3 ubiquitin ligase, can promote STAT1-mediated ISG expression and can induce viral 3C^pro^ degradation [111]. These observations provide guidance for studies of interferon signaling and viral infection. The mechanisms by which picornaviruses inhibit cellular type I IFN and the strategies by which host cells adopt a defense system are complicated.

The 3C^pro^ of another, HAV, has the capability to induce proteolysis of MAVS by the viral 3ABC precursor. The cleavage of this mitochondrially localized adaptor of RIG-I and MDA5 disrupts activation of IRF3 and abolishes type I IFN responses. The protease activities of 3C^pro^ and the transmembrane domain of 3A are both necessary for the proteolysis of MAVS [112,113]. For another precursor, HAV 3CD protease-polymerase disrupts TLR3 signaling by degrading TRIF (Figure 6) and this degradation is not dependent on only the protease activity of 3C^pro^ but also requires the 3D sequence to modulate the substrate specificity of 3C^pro^ [114]. NEMO, a bridging adaptor protein for the activation of NF-κB, is another substrate of HAV 3C^pro^ at the Q304 residue (Table 4) [115]. NEMO is also cleaved by FMDV 3C^pro^ at the Gln-383 residue but not by the 3C^pro^ of EV 71 [116].

Except for the cleavage of NEMO by 3C^pro^ [116], the L^pro^ of FMDV is also associated with the degradation of p65/RelA and decrease in IRF3/7 expression to suppress the immune response [117,118]. Subsequently, a study has shown that 3C^pro^ induces the degradation of karyopherin α1 (KPNA1) to antagonize interferon via the JAK-STAT signaling pathway by blocking the nuclear translocation of STAT1/STAT2 [119]. In CV, 3C^pro^ exhibits the capability of cleaving the proline-rich region of MAVS and two terminal domains of TRIF to escape host immunity (Figure 6) [120].

In addition to viral and host proteins, microRNAs (miRNAs) is also involved in posttranscriptional gene expression regulation and it has been confirmed that miRNAs play significant roles in the interaction between virus and host. The 3C^pro^ of EV71 downregulates miR-526a to suppress the RIG-I-dependent innate immune response [121].

A novel model shows that CVB3 manipulates transactive response DNA-binding protein-43 (TDP-43) to regulate viral pathogenesis. Normally, TDP-43 regulates RNA metabolism in the nucleus as a heterogeneous ribonucleoprotein (hnRNP), and a pathophysiological link between TDP-43 and amyotrophic lateral sclerosis (ALS) has been established [122]. When cells are infected by CVB3, 2A^pro^ induces translocation of TDP-43 from the nucleus to the cytoplasm. TDP-43 is then cleaved by 3C^pro^ at Q327, generating two fragments. The *C*-terminal fragment of TDP-43 is rapidly degraded, whereas the *N*-terminal product negatively regulates the function of native TDP-43 [123]. These processes benefit viral replication. The cleavage of full-length TDP-43 is closely related to neurodegeneration. Studies of 3C^pro^ and TDP-43 will provide information regarding the pathogenesis of CV.

The development of effective immune evasion mechanisms is beneficial to viral infection. Evidence has shown that picornaviruses adopt multiple mechanisms to suppress host antiviral responses. EV encodes 2A^pro^ and 3C^pro^ for polyprotein processing, whereas FMDV encodes L^pro^ and 3C^pro^. These proteins with proteolytic activity target and cleave certain cellular proteins to down-regulate the type I IFN response, pro-inflammatory cytokines and ISG induction. HAV encodes 3C^pro^ for its polyprotein processing. The multipronged cleavage of innate immune proteins is a remarkable example of picornaviral evolution through 3C^pro^ and its 3ABC and 3CD precursors, and 3C^pro^ can bind to and cleave different cellular molecules to interfere with antiviral signaling pathways to evade host immune response.

## 6. Treatment

### 6.1. Inhibitors of Picornaviral 3C^pro^

Picornaviral 3C^pro^ serves as an attractive target for antiviral drugs against picornaviruses because it plays an essential role in the virus replication cycle and it does not have cellular homologues. Some small peptides and a ketone-containing tripeptide, known as peptide aldehydes, can inhibit 3C^pro^ [124,125]. The peptidyl aldehyde NK-1.8 k targets 3C^pro^ to inhibit the replication of different EV71 strains and one EV68 strain [126].

Peptidyl Michael acceptors have been used to develop 3C^pro^ inactivators for inhibiting viral replication [127,128,129]. As a potent irreversible inhibitor of HRV 3C^pro^, AG7088 contains an α,β-unsaturated ester at P1′ that serves as a Michael acceptor, a lactam ring at P1, a fluoro-phenylalanine at P2, a Val at P3 and a 5-methyl-3-isoxazole. AG7088 binds with the active site Cys residue to influence the functionality of 3C^pro^ [130]. Through a series of cell-based protease assays, the activity spectra of the 3C^pro^ inhibitors AG7088 and SG85 have been found to include inhibition of CVB3, EV71, PV, and HRV14 3C^pro^s. However, these inhibitors have no effect on the 3C^pro^ of EMCV [131,132]. The inhibition of HAV replication by AG7088 is strain-dependent [133]. As an analogue of AG7088, AG7404 has been demonstrated to inhibit HRV, PV and several EV types [134].

Although covalent binding results in strong inhibition, the electrophilic nature of irreversible 3C^pro^ inhibitors may increase off-target safety risks due to potential interactions with host cysteine proteases [135]. Thus, exploration of non-covalent inhibitors of picornaviral 3C^pro^s has already begun. Non-covalent inhibitors of RV 3C^pro^ and CVB3 3C^pro^ have been identified through fragment screening and FRET-based enzyme assay screening [136]. The chemical modification from water-insolubility to water-solubility shows a positive effect. Indeed, water-insoluble 3C^pro^ inhibitors of CV B3 dissolved in 100% dimethyl sulfoxide (DMSO) have been reported to inhibit viral proliferation and to decrease myocardial damage and mortality in a chronic myocarditis model [137,138]. With the exception of organic solvents, organic reagents exhibit potential as 3C^pro^ inhibitors. Interaction between the cyanohydrins moiety and 3C^pro^ catalytic site exhibits high selectivity and improved inhibitory activity [139].

### 6.2. Broad-Spectrum Inhibitors of 3C^pro^ and 3CL^pro^

3C^pro^ and 3C-like protease (3CL^pro^) encoded by the *Picornaviridae*, *Caliciviridae*, and *Coronaviridae* families, are necessary for virus replication. Sharing a typical chymotrypsin-like fold, an extended binding site, a Cys-His-Glu/Asp catalytic triad (EV, CV 3C^pro^ and NV 3C^pro^) or Cys-His dyad (SARS-CoV 3CL^pro^), and a cleavage preference at the Gln-Gly (P1-P1′) junction, 3C^pro^ and 3CL^pro^ have been investigated as broad-spectrum antiviral targets, though 3C^pro^ is a monomer and 3CL^pro^ is a dimer [140]. The typical inhibitor of picornavirus 3C^pro^ AG7088 fails to inhibit SARS-CoV 3CL^pro^ due to subtle differences in the active site structures [141]. However, a modified AG7088 shows good inhibition of 3CL^pro^ and no inhibition of 3C^pro^ [142]. Using high-throughput screening, one compound that contains a central dihydropyrazole ring and four surrounding rings has been identified as equally inhibiting both the PV 3C^pro^ and CoV 3CL^pro^ [143]. By designing tripeptidyl transition state inhibitors and mimics, highly potent inhibitors that function against picornaviruses, coronaviruses and noroviruses have been discovered with minimal or no ability to inhibit a panel of proteases such as trypsin, thrombin or α-chymotrypsin [144]. The first macrocyclic inhibitor of 3C^pro^ of CVB3, 3CL^pro^ of SARS-CoV and 3CL^pro^ of noroviruses has been reported, with inhibitory activities of 1.8, 15.5 and 5.1 μM, respectively [145]. Research on picornaviral 3C^pro^ can provide a reference for other viruses.

### 6.3. Natural Medicine

Some extracts of plants and microbes have also been found to inhibit the proteolytic activity of 3C^pro^. As a member of the pyanonaphthoquinone family, the natural product (−)-thysanone has been reported to inhibit HRV 3C^pro^ with an IC_50_ value of 47 μM [146]. Through synthesis of analogues and the biological evaluation of (−)-thysanone, the 2-methylene analogue of (−)-thysanone has lost the capacity to inhibit HRV 3C^pro^ [147]. However, 9-deoxy analogues of (−)-thysanone display better inhibitory ability than the natural product [148]. Studies of herbs have demonstrated that flavonoids, such as fisetin and rutin, can inhibit the enzymatic activity of EV 71 3C^pro^ in a dose-dependent manner with IC_50_ values of 85 and 110 μM, respectively [149].

## 7. Conclusions

Due to their proteinase activities, 3C^pro^ and its precursor 3CD are involved in the processing of the polyprotein, but their catalytic efficiencies and specificities exhibit differences that are related to the different substrate-binding site residues in the 3C domain and the N-terminus of 3C^pro^. The RNA-binding activity of 3C^pro^ is important for initiating viral RNA synthesis. 3C^pro^ and its 3ABC, 3BC, 3BCD and P3 precursors can stimulate RNA synthesis at different levels. Due to the limited encoding capacity of picornaviruses, essential functions are performed by modulation of 3C^pro^ and its precursors.

The translation and replication of the picornaviral genome utilize the host translation machinery and the alteration and cleavage of host proteins lead to inhibition of host translation and transcription and nucleus-cytoplasm transport. 3C^pro^ and the 3CD precursor are involved in many steps of gene expression to hijack cellular resources and to generate an optimal intracellular environment for the virus life cycle. Disrupting mRNA circularization by cleaving PABP has been observed in PV, CV and HAV. After cleavage by 3C^pro^, some cellular factors and their fragments, such as eIF4G, PTB and PCBP2, function in distinct manners for the host cell and virus. These observations underscore the significant roles that 3C^pro^ plays in translation, replication, and the switch from translation to replication to guarantee viral replication. At the end of the replication cycle, the release and spread of virus are closely related to cell lysis and the formation of phosphatidylserine (PS) lipid-enriched vesicles [150]. 3C^pro^ not only induces cell death through caspase-dependent and caspase-independent pathways but also mediates fragmentation of MAP-4 and the Golgi apparatus. These findings clearly demonstrate the various mechanisms of 3C^pro^ that are required to subvert different processes in host cells to promote the viral life cycle.

Inhibition of host protein synthesis via cleavage of proteins required for transcription and cap-dependent translation can also block the production and transportation of antiviral proteins. In addition to innate immune responses, picornaviruses employ diverse mechanisms to suppress antiviral responses. There are some similarities and differences among various genera. With the help of precursors and the cooperation of other viral proteinases, 3C^pro^ has a crucial role in down-regulating type I IFN, pro-inflammatory cytokines and ISG induction. Picornavirus-infected cells engage multiple mechanisms. For example, the PARP9-DTX3L complex promotes ISG expression and induces degradation of viral 3C^pro^ to enhance the immune response. The cleavage of TDP-43 is a major molecular marker for some neurodegenerative diseases, and TDP-43 cleavage by the 3C^pro^ of CVB3 provides a novel model for viral pathogenesis research.

Understanding the structure and functions of 3C^pro^ provides accurate information for the design of broad-spectrum anti-picornaviral inhibitors. The development of irreversible inhibitors and the discovery of non-covalent inhibitors of 3C^pro^ will accelerate the treatments for infection by picornaviruses and other viruses encoding 3CL^pro^. Due to the limitations involved with using killed whole virus, recombinant empty capsids, which are structural and immunogenic mimics of virus particles, have become a research hotspot for an alternate vaccine. The development and the production of empty capsids is dependent on 3C^pro^ levels [3,151]. 3C^pro^ is well known to be a crucial proteinase for processing polyprotein and RNA-binding protein in viral replication. However, there is little information to date regarding the molecular mechanism by which 3C^pro^ blocks various cellular pathways. Comparison of 3C^pro^ functions in the life cycles of the host cell and virus can provide more insight into the pathogenesis and regulatory mechanisms of picornavirus. Comparison of 3C^pro^ functions in different genera reveals that this proteinase still maintains distinct functions in these genera. In conclusion, a mechanistic understanding of 3C^pro^ will have a potential future impact on antiviral treatment.

## Figures and Tables

**Table 1:** Cleavage of cellular transcription factors by 3C^pro^.

Classification of RNA Polymerases	Host Cell Protein	Virus	Functions	Cleavage Site	Reference
I	TAF_110_	PV	Regulates of transcriptional initiation	IQLQ_265_…ACAQ_805_G	[49]
II	TBP	PV	Involved in three RNA polymerase-mediated transcriptions and responsible for DNA binding	AAAVQQ_104_STSQQA	[50]
	CREB-1	PV	Binds to DNA elements for induction by cAMP	GQYIAITQ_172_GGAIQL	[51]
	Oct-1	PV	Binds to the octamer sequence ATGCAAAT and activates the promoters of genes for some small nuclear RNAs	KLGFTQ_329_GDVGLA (Speculated)	[52]
	P53	PV	Functions as a typical sequence specific transcription activator and a tumor suppressor	Not clear	[53]
III	TFIIIC	PV	Binds to the B-box internal promoter element to process tRNA transcription	TSQPPVPQ_732_GEAEED	[54]
Other factors	CstF64	EV 71	Polyadenylation of host mRNA	MQASMQ_251_GGVPAPGQMP	[56]
	Histone 3	FMDV	DNA binding and involved in the structure of chromatin in eukaryotic cells	GKAPRKQL_120_ATKAAR	[55]

**Table 2:** Cleavage of translation-related proteins by 3C^pro^.

Host Cell Protein	Virus	Functions	Cleavage Site	Reference
eIF4A I	FMDV	Binds capped mRNA to the 40S ribosomal subunit and unwinds double-stranded RNA	CIGGTNVRAE_143_VQKLQMEA	[63]
eIF4G I	FMDV	Brings mRNA to the 40S ribosome in translation initiation	RRSQQGPRKE_712_PRKIIATVL	[61]
eIF5B	PV	Positions the initiation methionine tRNA on the start codon of the mRNA	LCAAVEVMEQ_478_GVPEKEET	[64]
	CV			
	HRV			
G3BP1	CV	RNA-binding protein that interacts with Ras-GAP	EAGEQ_325_GDIEP	[70]
PCBP2	HAV	Translational activation and control of gene expression	IGRQ_306_GAKI (Speculated)	[66]
	PV		AMQQ_253_SHFP…IGRQ_306_GAK	[65]
PABP	PV	Involved in poly (A) shortening and translation initiation	Not clear	[67]
	HAV		Not clear	[68]
	EMCV		VRPPAAIQ_437_GVQAGA	[69]
Sam68	FMDV	Involved in cellular differentiation and proliferation	C-terminal portion	[71]

**Table 3:** *Picornavirus*-induced alterations to the nuclear pore complex.

Genus	Virus	Viral Protein	Host Protein	Reference
*Enterovirus*	Rhinovirus	2A^pro^	Nup 62	[77]
		3C^pro^/3CD	Nup 153, Nup 214, Nup 358	[78]
		3C^pro^	Nup 62	[78]
	Poliovirus	2A^pro^	Nup 62, Nup 98, Nup 153	[76]
*Cardiovirus*	Encephalomyocarditis virus	Leader protein	Nup 62, Nup 153, Nup 214	[81]
	Theiler’s murine encephalomyelitis virus	Leader protein	Nup 62, Nup 98	[82]

**Table 4:** Cleavage of innate immune-related proteins by 3C^pro^.

Virus	Host Factors	Cleavage Site	Reference
FMDV	NEMO	LSSPLALPSQ_383_RRSPPEEPPD	[116]
EV71	IRF7	GDLLLQAVQQ_189_SCLADHLLTA	[108]
	TRIF	TEGSAGPQ_312_SLPLPILEP	[104]
	TAK1	KNQAKQQ_360_SESGRL	[109]
	TAB2	SISDGQLQ_113_GGQSNSEL	[109]
CVB3	MAVS	SYPMPVQETQ_148_APESPGENSEQ	[120]
	TRIF	Q190, Q653, Q659, Q671, Q702 (potential)	[120]
HAV	NEMO	METVPVLKAQ_304_ADIYKADFQA	[115]
	TRIF	SDWSQ_190_GCSLR…EQSQ_554_HLDGER	[114]
	MAVS	LASQ_428_VDSP…YKSE_463_GTFG (potential)	[113]
